# Cranio-caudal and medio-lateral navicular translation are representative surrogate measures of foot function in asymptomatic adults during walking

**DOI:** 10.1371/journal.pone.0208175

**Published:** 2018-12-05

**Authors:** Patric Eichelberger, Johannes Pohl, Theo Jaspers, Matteo Ferraro, Fabian Krause, Heiner Baur

**Affiliations:** 1 Bern University of Applied Sciences, Department of Health Professions, Division of Physiotherapy, Bern, Switzerland; 2 Graduate School for Cellular and Biomedical Sciences, University of Bern, Bern, Switzerland; 3 University Hospital Bern, Inselspital, Department of Orthopedic Surgery, Bern, Switzerland; Virginia Commonwealth University, UNITED STATES

## Abstract

**Introduction:**

The translation of the navicular bone is thought to be a representative surrogate measure to assess foot pronation and hence foot function; however, it is not known how it is related to multi-segment foot kinematics.

**Methods:**

Cranio-caudal (NCC) and medio-lateral (NML) navicular translation and multi-segment foot kinematics from the Oxford Foot Model (OFM) were simultaneously assessed during the stance phase of walking in 20 healthy adults. Relationships to forefoot to hindfoot (FFtoHF), hindfoot to tibia (HFtoTBA) and global hindfoot (HFL) motion were explored by cross-correlations at zero phase shift.

**Results:**

FFtoHF sagittal, transversal and frontal plane angles showed median cross correlations of -0.95, 0.82 and 0.53 with NCC and of 0.78, -0.81 and -0.90 with NML. HFtoTBA transversal and frontal plane angles had correlations of 0.15 and 0.74 with NCC and of -0.38 and -0.83 with NML. The HFL frontal plane angle showed correlations of 0.41 and -0.44 with NCC and NML, respectively.

**Discussion:**

The strongest relationships were found between FFtoHF sagittal plane angles and NCC and between FFtoHF frontal plane angles and NML. However, cranio-caudal and medio-lateral navicular translation seem to be reasonable surrogates for the triplanar motion between the fore- and hindfoot. The medial longitudinal arch dropped and bulged medially, while the forefoot dorsiflexed, abducted and everted with respect to the hindfoot and vice-versa. The lower cross-correlation coefficients between the rear foot parameters and NCC/NML indicated no distinct relationships between rearfoot frontal plane and midfoot kinematics. The validity of rearfoot parameters, like Achilles tendon or Calcaneal angle, to assess midfoot function must be therefore questioned. The study could also not confirm a systematic relationship between midfoot kinematics and the internal/external rotation between the hindfoot and the tibia. The measurement of navicular translation is suggested as an alternative to more complex multi-segment foot models to assess foot function.

## Introduction

The human foot represents a complex biomechanical system [1] consisting of numerous bones and ligaments and is actuated through various intrinsic and extrinsic muscles, building a dynamic link between the body and the ground [2]. As such, the foot enables a harmonious coupling between the body and the environment for successful upright locomotion [2]. For being flexible to absorb power and adapt to the ground, on the one hand, and being stiff to generate propulsion power during push-off, on the other hand, the pronation-supination mechanism of the foot is essential [3]. Foot pronation is basically understood as inward rolling of the foot and, due to the oblique orientation of the subtalar joint axis [4], represents a triplanar movement composed of rearfoot eversion and forefoot abduction and dorsiflexion [3, 5]. Quasi-static experiments [6–8], in vitro walking simulation [9] and studies on walking gait [1, 10] provide evidence that the midfoot plays a central role for foot pronation due to the large ranges of motion at the talonavicular joint and the navicular-medial cuneiform articulation. A marker at the navicular bone provides valuable information for midfoot kinematics [11]. Assessment of the navicular mobility in terms of cranio-caudal (NCC) and medio-lateral (NML) translation of the navicular tuberosity originates from the domain of clinical evaluation of the foot and is thought to be representative for foot pronation [12, 13]. The concept goes back to the ideas of navicular drop and drift, the vertical or medial excursion of the navicular tuberosity when going from a subtalar neutral position into a relaxed calcaneal position, which were proposed as quasi-static estimates for the amount of foot pronation and to provide further insights into talonavicular joint mechanics [14–16]. Navicular translation during dynamic tasks can be assessed relatively easily in practice with a set of four markers [17] compared to multi-segment foot kinematics, whose measurement is more complex and time-consuming and which require dedicated motion capture infrastructure. Up to date, the evidence regarding if and how NCC and NML are systematically associated with multi-segment foot kinematics is poor. The study therefore aimed to explore how the navicular translation, quantified by cranio-caudal and medio-lateral navicualar displacement, is related to multi-segment foot kinematics during the stance phase of walking.

## Materials and methods

### Subjects

The cross-sectional study was carried out on active and healthy adults (18-60 years old; min. two sport activities per week) who were free from any musculoskeletal complaints or acute infections, had had no surgery on the lower extremities during the previous 12 months and did not perform any intensive training session the day before the examination. All participants signed an informed consent and ethics approval for the study was retrieved from the Ethics Committee of the Canton of Bern (KEK-No. 052/15). Twenty-two participants were included and examined but only the data from 20 persons were eligible for further analysis due to data acquisition errors in two cases. One subject was additionally excluded from further statistical analysis in addition because this person presented an atypical and non-representative walking pattern, characterized by making initial contact with the midfoot and a lacking heel rocker phase [4]. Subjets’ antropometric data is presented in Table 1.

**Table 1:** Subjets’ anthropometric data [Mean (SD)].

Parameter	Males	Females
n	13	6
Foot length (mm)	268 (10)	242 (13)
Age (years)	31.2 (7.3)	26.8 (4.2)
Body height (cm)	181 (5)	170 (8)
Body mass (kg)	76.5 (8.6)	64 (12.2)
Walking speed (km h^−1^)	4.2 (0.4)	4.5 (0.4)

### Measurement procedure

**Fig 1:**
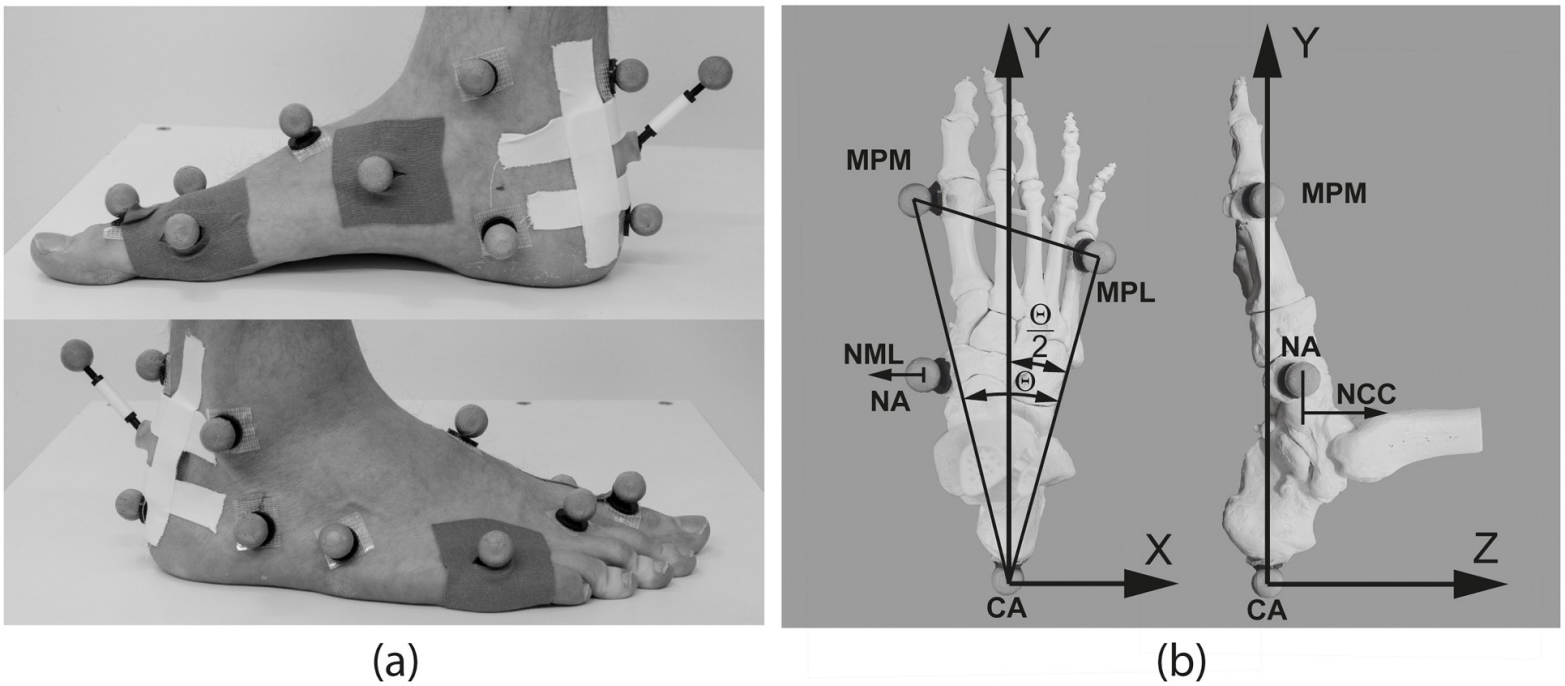
Skin-surface markers used for measuring right foot kinematics (a) and description of the 4-marker foot model (b). (**a**) Markersets for the OFM and the 4-marker foot model applied to the right foot. The markers at the middle of the dorsal calcaneous (CA), the lateral caput of the 5^th^ metatarsal bone (MPL) and the medial caput of the 1^st^ metatarsal bone (MPM) were shared by both models. The marker at the tuberosity of the navicular bone (NA) was applied in addition for the 4-marker foot model. (**b**) 4-marker foot model to measure cranio-caudal (NCC) and medio-lateral (NML) navicular displacement.

Foot kinematics were measured using a three-dimensional motion capture system with 10 cameras (Vicon Motion Systems, Ltd, Oxford, UK; 8x Bonita 3 and 2x Bonita 10 cameras at 200 Hz) that provided a measurement volume of (4 x 1.5 x 1.5) m^3^). Ground reaction forces were measured with a force plate (AMTI OR6, AMTI Inc., Watertown, USA; sampling frequency 1 kHz) and served for initial contact and toe off identification (threshold at 25 N) to subsequently extract the kinematics during stance. While the participants were walking at self-selected speed, the kinematics of the right foot were simultaneously captured by a set of four markers to quantify the navicular mobility in terms of cranio-caudal (NCC) and medio-lateral translation (NML) of the navicular tuberosity (Fig 1b) [17] and by the Oxford Foot Model (OFM) [18] to measure forefoot to hindfoot (FFtoHF), hindfoot to tibia (HFtoTBA) and global hindfoot rotations (HFL) (Fig 1). The foot’s longitudinal axis was the bisecting line of the MPM-MPL-CA triangle. The X-Y plane was calibrated to be parallel with the foot’s planar surface based on a static bipedal standing trial and defined via the plane spanned by MPM-MPL-CA markers. NML was measured parallel to the x-axis and NCC was measured parallel to the z-axis. The 3D rotations output by the OFM were reported as Euler angle sequence of 1) sagittal plane rotations (plantar-/dorsalflexion), 2) transversal plane rotations (adduction/abduction for FFtoHF angles; internal/external rotation for HFtoTBA and HFL angles) and 3) frontal plane rotations (pro-/supination for FFtoHF angles; inversion/eversion for HFtoTBA and HFL angles). The software Vicon Nexus version 1.8.5 was used for measurement and version 2.5 for 3D point reconstruction, marker labelling, gait event detection and in the case of the OFM to calculate the model outputs. The Vicon PlugInGait Lowerbody model is a prerequisite for the OFM and the OFM markerset was applied in addition to the PluginGait Lowerbody markerset. The PlugInGait Lowerbody model is part of Nexus by default and the OFM is available as plug-in from Vicon. The joint kinematics from the PlugInGait Lowerbody model and the OFM were calculated within Nexus while the navicular translation parameters were calculated with custom-made software in Matlab (Version 2017a, The MathWorks Inc., Natick, USA). The .c3d file format (www.c3d.org) and the btk-Toolkit [19] were used to access the measurement data from Matlab. Spherical skin surface markers with a diameter of 16 mm were used for the PluginGait Lowerbody model and of 14 mm diameter for the Oxford and navicular mobility foot models. Using smaller markers for the measurement of foot kinematics was necessary to properly resolve the intrinsic foot motion within the given measurement volume. An experienced physical therapist palpated the anatomical landmarks and applied the reflective markers by means of double-face adhesive tape while the participants were in bipedal upright standing pose (Fig 1).

### Data analysis

Data analysis was carried out in Matlab and included the extraction and normalization of stance phase time-series (0-100% stance phase; 200 samples/stance phase) and averaging among the trials from each participant. Between 6 and 15 walking trials from each participant were averaged to retain representative stance phase kinematics for further analysis. An analysis was made of how the two navicular mobility measures related to each other and to the forefoot to hindfoot (FFtoHF), the hindfoot to tibia (HFtoTBA) and the hindfoot to laboratory (HFL) angles. Translational (NCC, NML) and rotational (FFtoHF, HFtoTBA, HFL) displacements from initial contact to toe off were considered in the analysis. The following relationships were explored further qualitatively by displacement-displacement plots and quantitatively by cross-correlation coefficients at zero phase shift (*X*_*c*_) [5, 10]: (i) NCC to NML, (ii) NCC and NML to FFtoHF plantar-/dorsiflextion, adduction/abduction and pro-/supination, (iii) NCC and NML to HFtoTBA internal/external rotation and inversion/eversion, and (iv) NCC and NML to HFL inversion/eversion. The cross-correlation coefficients were calculated with the cross-correlation function xcorr() from Matlab’s Signal Processing Toolbox (normalization option ‘coeff’ to normalize the cross-correlation sequence so that *X*_*c*_ ∈ [−1, 1]). In accordance with Pohl et al. [5], the following conventions were used for interpreting the degree of the relationships between the model outputs:

- Strong: −1 ≤ *X*_*c*_ ≤ −0.8 and 0.8 ≤ *X*_*c*_ ≤ 1
- Moderate: −0.8 < *X*_*c*_ ≤ −0.3 and 0.3 ≤ *X*_*c*_ < 0.8
- Weak: −0.3 < *X*_*c*_ < 0.3

Compared to Pohl et al. [5], who considered ≥ 0.7 (or ≤ −0.7) as strong, the thresholds used in this study for classifying a relationship as strong were more restrictive. The ranges of motion (RoMs) must be taken into account when interpreting relationships based on cross-correlation coefficients because ranges of motion [10] below measurement errors may lead to large cross-correlation values and hence to an overinterpretation that two kinematic time-series are strongly related even if one or both movements were not substantial. We therefore adopt an error threshold of 3° mentioned by Wolf et al. [10] for the angular displacements and use an error threshold of 2 mm for the navicular displacements (RMSE estimate of 1 mm [17]), which the RoMs must exceed to classify a relation as valid.

In addition to looking at the whole stance phase, the analysis of the relationships between the various foot kinematic measures was subdivided into phases of power absorption and generation ($X_{c_{\text{Abs}}}$ and $X_{c_{\text{Gen}}}$, respectively) based on the anterior-posterior ground reaction force, which represents the two phases related to the body center of mass in the direction of progression. This was done to explore the relationships more closely to the two fundamental functional requirements of foot function, on the one hand, providing flexibility for shock absorption and balance and, on the other hand, stiffness for effective propulsion during push-off [3, 4]. The two phases were discriminated by the direction of the anterior-posterior ground reaction force component, which turns from posterior to anterior at around 50% of stance as the body center of mass is moved from behind to the front of the foot fixed to the ground during normal walking [4].

## Results

**Fig 2:**
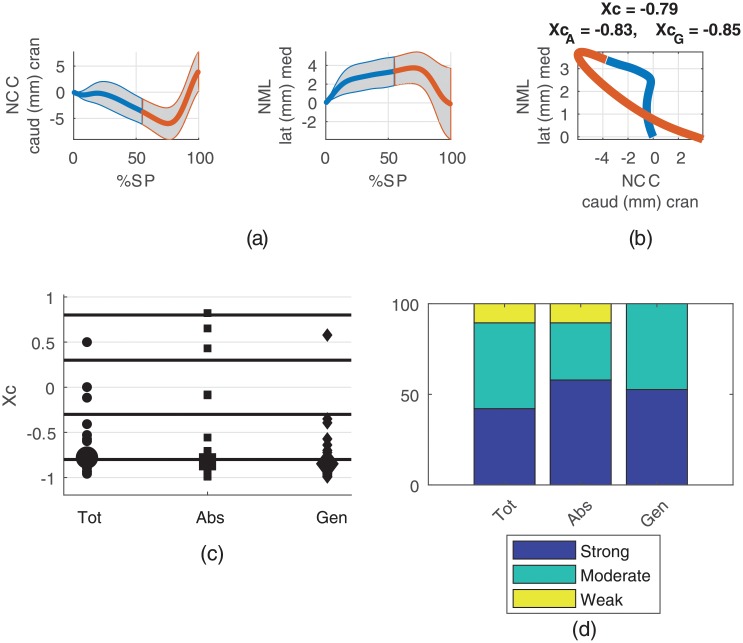
Relationships between cranio-caudal (NCC) and medio-lateral (NML) navicular displacement. (**a**) Averages among participants with standard deviations for NCC and NML during the stance phase (SP). Corresponding ranges of motion are given in Table 2. The blue parts represent the absorption and the red parts the generation phases. (**b**) Displacement-displacement diagram of mean NML versus mean NCC with median cross-correlation coefficients for the whole stance phase (*X*_*c*_), the absorption phase ($X_{c_{\text{A}}}$) and the generation phase ($X_{c_{\text{G}}}$) in the title. (**c**) Scatter plots of cross-correlation coefficients for the whole stance phase (Tot), the absorption phase (Abs) and the generation phase (Gen) from all participants for the relation between NCC and NML. Large markers represent the medians. (**d**) Distribution of the cross-correlation coefficients among the strong, moderate and weak classes (values given in Table 3).

**Fig 3:**
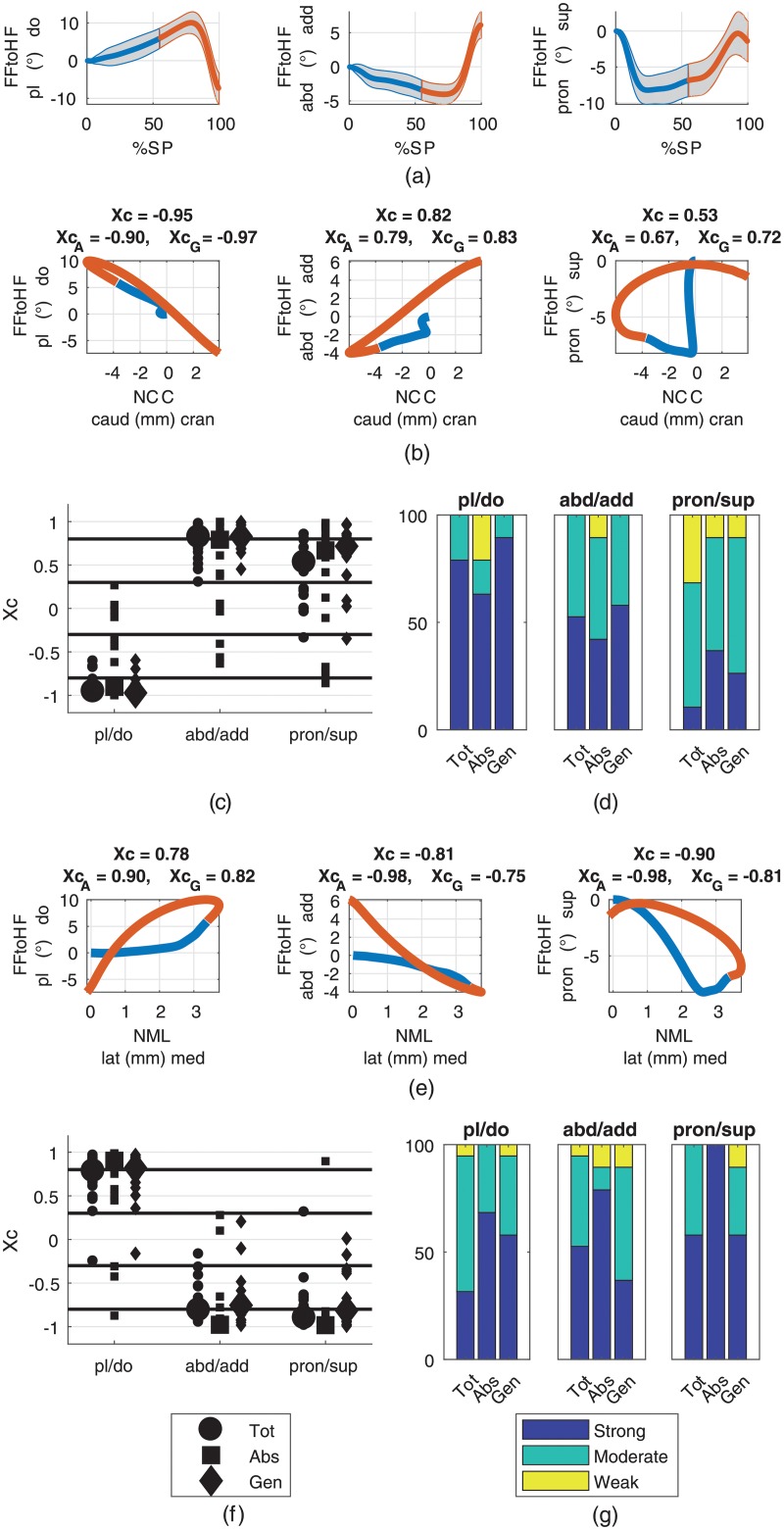
Relationships of cranio-caudal/medio-lateral navicular displacements (NCC/NML) to forefoot to hindfoot (FFtoHF) rotations. (**a**) Averages among participants with standard deviations for FFtoHF plantar/dorsiflexion, adduction/abduction and pronation/supination during the stance phase (SP). Corresponding ranges of motion are given in Table 2. The blue parts represent the absorption and the red parts the generation phases. (**b**), (**e**) Displacement-displacement diagrams of mean FFtoHF rotations versus mean NCC (b) and mean NML (e) with median cross-correlation coefficients for the whole stance phase (*X*_*c*_), the absorption phase ($X_{c_{\text{A}}}$) and the generation phase ($X_{c_{\text{G}}}$) in the titles. (**c**), (**f**) Scatter plots of cross-correlation coefficients for the whole stance phase (Tot), the absorption phase (Abs) and the generation phase (Gen) from all participants for the relationships presented in rows 2 and 4. Large markers represent the medians. (**d**), (**g**) Distribution of the cross-correlation coefficients among the strong, moderate and weak classes (values given in Table 3).

**Fig 4:**
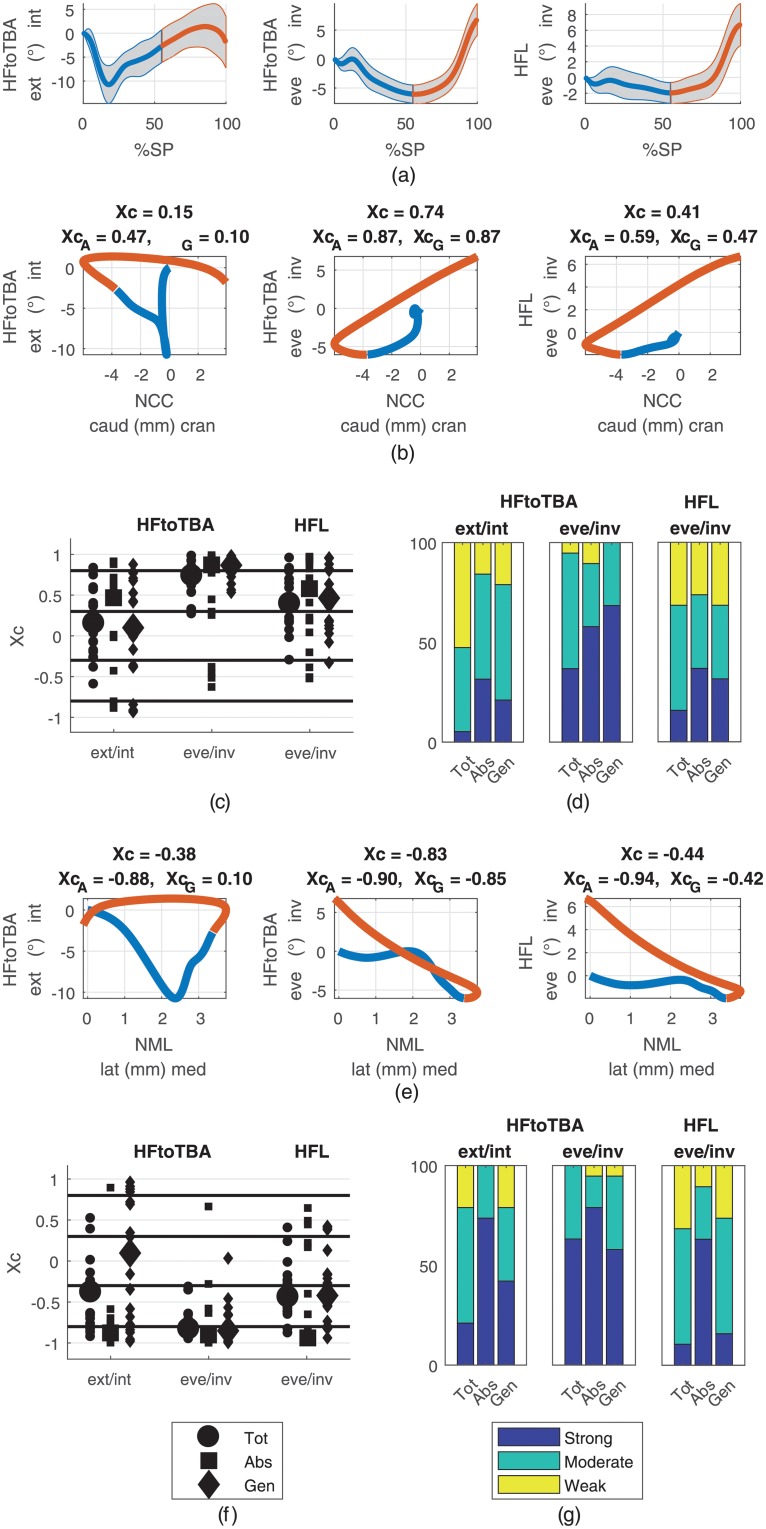
Relationships of cranio-caudal/medio-lateral navicular displacements (NCC/NML) to hindfoot to tibia (HFtoTBA) and global hindfoot (HFL) rotations. (**a**) Averages among participants with standard deviations for HFtoTBA internal/external rotation and inversion/eversion and for HFL inversion/eversion during the stance phase (SP). Corresponding ranges of motion are given in Table 2. The blue parts represent the absorption and the red parts the generation phases. (**b**), (**e**) Displacement-displacement diagrams of mean HFtoTBA and mean HFL rotations versus mean NCC and mean NML with median cross-correlation coefficients for the whole stance phase (*X*_*c*_), the absorption phase ($X_{c_{\text{A}}}$) and the generation phase ($X_{c_{\text{G}}}$) in the titles. (**c**), (**f**) Scatter plots of cross-correlation coefficients for the whole stance phase (Tot), the absorption phase (Abs) and the generation phase (Gen) from all participants for the relationships presented in rows 2 and 4. Large markers represent the medians. (**d**), (**g**) Distribution of the cross-correlation coefficients among the strong, moderate and weak classes (values given in Table 3).

Transition from the power absorption to the power generation phase occurred at 55 (SD 2.7)% stance phase which corresponded well with the initiation of heel lifting (see S1 Fig in the supporting information). The ranges of motion, calculated as the difference between minimum and maximum value for stance, absorption and generation phases are presented in Table 2. Fig 2 presents the relationships between the cranio-caudal and medio-lateral navicular displacements, which showed mean RoMs for the stance phase of 10.5 (SD 3.5) mm and 5.7 (SD 2.4) mm, respectively. The median cross-correlation between them was -0.79 (-0.83/-0.85) ($X_{c_{\text{Abs}}}$/$X_{c_{\text{Gen}}}$). The relationships of the cranio-caudal and medio-lateral navicular displacements to the forefoot-hindfoot and to the hindfoot kinematics are illustrated in Figs 3 and 4, respectively. The RoMs among the stance phase of the forefoot to hindfoot dorsi-/plantarflexion, abduction/adduction and pronation/supination were 17.7 (SD 3.2), 10.5 (SD 2.2) and 9.8 (SD 2.0) degree, respectively. The respective median cross-correlations with the cranio-caudal navicular displacement were -0.95 (-0.90/-0.97), 0.82 (0.79/0.83) and 0.53 (0.67/0.72) and with the medio-lateral navicular displacement 0.78 (0.90/0.82), -0.81 (-0.98/-0.75) and -0.90 (-0.98/-0.81). The RoMs among the stance phase of the hindfoot to tibia external/internal rotation and eversion/inversion were 14.2 (SD 4.0) and 13.2 (SD 3.0) degree, respectively. They showed median cross-correlations of 0.15 (0.47/0.10) and 0.74 (0.87/0.87) with the cranio-caudal and -0.38 (-0.88/0.10) and -0.83 (-0.90/-0.85) with the medio-lateral navicular displacement. The RoM of the global hindfoot eversion/inversion was 9.2 (SD 2.6) degree and it showed median cross-correlations of 0.41 (0.59/0.47) and -0.44 (-0.94/-0.42) with the cranio-caudal and medio-lateral navicular translation, respectively. Table 3 summarizes the cross-correlation coefficients and their distributions among the weak, moderate and strong correlation bands for all examined model output pairs examined. Outputs from the four marker foot model and the Oxford Foot Model for all stance phases included in the analysis are provided as supporting information (S1 and S2 Data, respectively).

**Table 2:** Means and standard deviations of observed ranges of motion (minimum to maximum) for the whole stance phase (RoM), the absorption phase (RoM_Abs_) and the generation phase (RoM_Gen_) [Mean (SD)].

	RoM	RoM_Abs_	RoM_Gen_
FFtoHF pl/do (°)	17.7 (3.2)	6.6 (2.4)	17.7 (3.2)
FFtoHF abd/add (°)	10.5 (2.2)	3.6 (1.3)	10.5 (2.2)
FFtoHF pron/sup (°)	9.8 (2.0)	8.6 (2.0)	7.3 (2.8)
HFtoTBA ext/int (°)	14.2 (4.0)	11.7 (4.3)	5.9 (2.4)
HFtoTBA eve/inv (°)	13.2 (3.0)	7.0 (1.7)	13.1 (3.0)
HFL eve/inv (°)	9.2 (2.6)	2.9 (1.2)	8.9 (2.5)
NCC (mm)	10.5 (3.5)	4.6 (2.0)	10.2 (3.6)
NML (mm)	5.7 (2.4)	3.6 (1.3)	4.3 (3.1)

**Table 3:** Median cross-correlation coefficients and percentages of strong (S), moderate (M) and weak (W) classifications for the stance, absorption and generation phases of all examined relationships. FFtoHF: forefoot to hindfoot rotations; HFtoTBA: forefoot to tibia rotations; HFL: hindfoot to laboratory rotations; NCC: cranio-caudal navicular motion; NML: medio-lateral navicular motion.

			Stance				Absorption				Geneneration			
			Xc	S (%)	M (%)	W (%)	Xc	S (%)	M (%)	W (%)	Xc	S (%)	M (%)	W (%)
FFtoHF	NCC	pl/do	-0.95	78.9	21.1	0.0	-0.90	63.2	15.8	21.1	-0.97	89.5	10.5	0.0
		abd/add	0.82	52.6	47.4	0.0	0.79	42.1	47.4	10.5	0.83	57.9	42.1	0.0
		pron/sup	0.53	10.5	57.9	31.6	0.67	36.8	52.6	10.5	0.72	26.3	63.2	10.5
	NML	pl/do	0.78	31.6	63.2	5.3	0.90	68.4	31.6	0.0	0.82	57.9	36.8	5.3
		abd/add	-0.81	52.6	42.1	5.3	-0.98	78.9	10.5	10.5	-0.75	36.8	52.6	10.5
		pron/sup	-0.90	57.9	42.1	0.0	-0.98	100.0	0.0	0.0	-0.81	57.9	31.6	10.5
HFtoTBA	NCC	ext/int	0.15	5.3	42.1	52.6	0.47	31.6	52.6	15.8	0.10	21.1	57.9	21.1
		eve/inv	0.74	36.8	57.9	5.3	0.87	57.9	31.6	10.5	0.87	68.4	31.6	0.0
	NML	ext/int	-0.38	21.1	57.9	21.1	-0.88	73.7	26.3	0.0	0.10	42.1	36.8	21.1
		eve/inv	-0.83	63.2	36.8	0.0	-0.90	78.9	15.8	5.3	-0.85	57.9	36.8	5.3
HFL	NCC	eve/inv	0.41	15.8	52.6	31.6	0.59	36.8	36.8	26.3	0.47	31.6	36.8	31.6
	NML	eve/inv	-0.44	10.5	57.9	31.6	-0.94	63.2	26.3	10.5	-0.42	15.8	57.9	26.3
NCC/NML			-0.79	42.1	47.4	10.5	-0.83	57.9	31.6	10.5	-0.85	52.6	47.4	0.0

## Discussion

The cross-sectional study on healthy adults investigated the relationship between cranio-caudal and medio-lateral navicular translation and selected multi-segment foot kinematics from the Oxford Foot Model during the stance phase of barefoot walking at a self-selected pace. This study aimed to validate the concept of using a minimal markerset to measure cranio-caudal and medio-lateral navicular translation, two surrogate parameters which seem promising to assess foot function during dynamic tasks [12, 13]. To draw the key conclusions from the data presented in this article it is important to note that the FFtoHF pronation from the OFM actually describes the frontal plane motion of the FF with respect to the HF and does not correspond with the clinical understanding of pronation, which is the triplanar combination of FF to HF dorsiflexion—abduction—eversion [3, 5]. Hence, cranio-caudal and medio-lateral translation can only be said to be associated with the clinical foot pronation when cross-correlations are high with all three FFtoHF outputs from the OFM.

### Relationship between cranio-caudal and medio-lateral navicular translation

The navicular translation parameters showed a pattern of caudal dropping while deviating medially up to a deflection point around 75% stance phase where the pattern turned into rising cranially and shifting laterally (Fig 2a). In addition, the NML showed a deflection around 20% stance phase, which was not apparent in NCC. Patterns similar to NCC were already observed by others, who studied the navicular drop during walking [20, 21]. The cranial rise exceeded the initial contact level at toe off. While the RoMs during absorption were similar, the RoM of NCC was more than twice as high as the RoM of NML during generation. The pattern is reflected in the displacement-displacement diagram (Fig 2b) and also in the cross-correlation coefficients which were primary classified as moderate and strong (Fig 2d) resulting in a relationship for the stance phase at the transition from moderate to strong (-0.79). Looking at the absorption and generation phases separately, the relationships became stronger. The mean RoMs were above the error threshold of 2 mm (Table 2), which indicates valid relationships.

### Relationships to forefoot to hindfoot kinematics

Sagittal and transversal plane kinematic waveforms of the forefoot with respect to the hindfoot showed similar patterns. The forefoot dorsiflexed while it abducted during approximately the first 75% of stance, and from then on it started to plantarflex and adduct until toe off. In contrast, this pattern did not become evident in the pronation/supination movement, where the maximum pronation was reached around 20% stance phase by subsequent forefoot supination. Nevertheless, all waveforms of the FFtoHF kinematics were consistent with observations that were also made with the Oxford Foot Model in healthy adults [22] and children [23]. The similarities of sagittal and transversal FFtoHF kinematics with the cranio-caudal and medio-lateral navicular displacements became evident qualitatively from the linear patterns in the displacement-displacement diagrams and quantitatively from the cross-correlation values (Fig 3, Table 3). A clear association could be observed between the FFtoHF plantar/dorsiflexion and the NCC displacement with median cross-correlation coefficients above 0.90 and more than 60% classified as strong in all phases (Table 3, Fig 3d) and a distinct linear pattern (Fig 3b left). The navicular bone dropped as the forefoot dorsiflexed and vice versa. The relationship with the NML displacement was close to the moderate to strong transition (0.78), which indicated that the navicular bone deviated medially as the forefoot dorsiflexed and vice-versa. The strength of the relationship between FFtoHF abduction/adduction and NCC and NML was similar (median cross-correlation around 0.80), although somewhat stronger for the absorption phase in the case of NML (-0.98), but which might be overestimated due to the relatively small RoM in FFtoHF abduction during absorption (Table 2). Basically, the forefoot abducted while the navicular bone dropped caudally and drifted medially and adducted while the navicular bone rised cranially and shifted laterally. The FFtoHF pronation/supination was only moderately linked to the cranio-caudal navicular displacement but showed a strong relationship to the medio-lateral navicular displacement with a median cross-correlation of -0.90 and more than 57.9% classified as strong in all phases (Table 3). The relationship was especially pronounced during the absorption phase (-0.98). Hence, the navicular bone deviated medially as the forefoot pronated and vice versa.

### Relationships to hindfoot kinematics

The waveform observed for the HFtoTBA external/internal rotation, which showed external rotation until around 20% stance phase followed by subsequent internal rotation, differed from those of HFtoTBA and HFL eversion/inversion which presented eversion until around the absorption/generation transition, followed by inversion. Nevertheless, both patterns were consistent with the literature [22]. HFtoTBA external/internal rotation was poorly related to NCC (0.15) and rather moderately to NML (-0.38), in which case the cross-correlation for the absorption phase was noticeably higher (-0.88) compared with the generation phase (0.10), although no linear pattern became apparent from the displacement-displacement diagram. This can be explained by the common shapes of NML and HFtoTBA external/internal rotation with distinct deflection points around 20% stance phase. Hence, the hindfoot rotated externally relative to the tibia (i.e. the tibia rotated internally relative to the hindfoot) as the navicular bone deviated medially during approximately the first 20% of the stance phase, but the navicular bone did not reverse the medial motion as the hindfoot reversed its direction of rotation towards internal during the remaining generation phase. For the generation phase relationships of HFtoTBA external/internal rotation, the cross-correlation values were heterogeneous among the participants and broadly spread from strong negative to strong positive (Fig 4f). Hence, the resulting median relationships were weak (0.10) although only for 21.1% of the participants the relationships were classified as weak (Fig 4g Table 3). HFtoTBA eversion/inversion was moderately (0.74), even if close to the strong transition, related to NCC and strongly related (-0.83) to NML, whereas in both cases the relationships were strong when considering the absorption and generation phases separately. The relationships of NCC and NML to the global hindfoot eversion/inversion were only moderate (0.44 and -0.44, respectively). The high cross-correlation of -0.94 for the absorption phase in the case of the relationship to NML cannot be classified as valid because the RoM of hindfoot inversion/eversion did not exceed the error threshold. That a strong relationship to HFtoTBA internal/external rotation was only found for the medio-lateral translation during absorption suggests that there is no systematic coupling between foot pronation and tibia rotation. The hindfoot eversion/inversion (i.e. the calcaneal angle) did not present any relationship to the navicular parameters but it turned out that the eversion/inversion of the hindfoot relative to the tibia (i.e. the Achilles tendon angle) led to relatively high cross-correlations. Hence, taking the frontal plane tibia motion in addition to the calcaneal angle into account seemed to highly increase the relationship between hindfoot motion and foot pronation. In fact, the frontal plane curves of the hindfoot and the hindfoot relative to the tibia did not represent substantial shape differences (Fig 4a). Therefore, the increase in cross-correlation coefficients must be attributed to the higher angular range due to the tibia motion and not to more similarity with the navicular translation curves. Hence, the relationship between navicular translation and hindfoot motion is suggested to be rather weak.

### Limitations

We included participants complying with the criteria of being asymptomatic but did not classify feet according to morphological criteria. This may explain the occasionally large spreads of cross-correlation coefficients. The results might differ in low- or high-arch, even though symptom-free, subgroups and the representativeness of the present study might be therefore limited. The results are limited to walking gait and might not be directly transferred to situations like walking on stairs or running. The cross-correlation analysis is limited to the fact that the coefficients capture only linear relationships between two signals. Hence, small values may result, even if two signals were strongly related but in a non-linear fashion (e.g. quadratic). When interpreting cross-correlation coefficients, which are in fact Pearson correlations in function of a phase shift between the two vectors under consideration, it must be born in mind that they are sensitive to the range of the vector values. Hence, larger cross-correlation coefficients might results from increased ranges of values rather than actually more similarity of curve shapes. In addition, the interpretation of the strength of relationships is dependent from the cross-correlation levels used for classification. Since cross-correlation coefficients are relative estimates on how much of the variance in a dependent variable can be explained by an independent variable, there is no absolute rule from when on a relationship can be said to be strong. However, we set this limit at 0.8 and are more restrictive in this respect than previous examinations [5]. Limitations due to soft tissue artifacts must always be borne in mind when applying skin surface marker based methods. Nevertheless, skin-based markers represent real life conditions in clinical gait analysis settings.

## Conclusion

Cranio-caudal and medio-lateral navicular translation seem to be reasonable surrogates for the triplanar motion between the fore- and hindfoot. However, frontal-plane motion seems only to be moderately represented by the cranio-caudal navicular translation. The strengths of cross-correlations indicate that a flattening and rising of the medial longitudinal arch (i.e. sagittal FFtoHF motion) is primary captured by the cranio-caudal navicular translation and that the forefoot-hindfoot twist (i.e. frontal plane FFtoHF motion) is primary captured by the medio-lateral navicular translation. Cranio-caudal and medio-lateral translation therefore seem to primary measure different aspects of foot function and should be evaluated separately. This statement is also supported by the NCC-NML relationship which was only found to be at the transition from moderate to strong. The relationship between navicular translation and hindfoot motion could not have been confirmed and therefore the Achilles tendon angle and the Calcaneal angle or calcaneal eversion excursion do not seem to be appropriate parameters to assess foot pronation. It is suggested that there is no legitimation to assess midfoot function by measurements at the hindfoot. Similar experiments for other modes of gait like running or stair walking might provide further insights into the relationship between navicular mobility and multi-segment foot kinematics. Taking foot type or symptomatic subgroups into account could strengthen the value of a follow-up study and contribute to a differentiated understanding of foot biomechanics and dysfunction. The approach with a minimal set of four markers provides potential for less complex 3D foot function assessment during dynamic tasks.

## Supporting information

S1 Fig Power absorption/generation transition. Means and standard deviations among participants (N = 19) of anterior-posterior (AP) and vertical ground reaction forces (Rx and Rz, respectively) normalized to the body weight force (BWF) and global plantar-/dorsiflexion angle of the foot (FPitch). The vertical lines indicate the transition of the AP ground reaction force from a posterior to an anterior direction (55 ± 2.7% stance phase) which was used to define the power absorption and power generation phases. This transition corresponds to the instance in time where lifting the heel from the ground was initiated.
(PDF)

S1 Data 4-Marker Foot Model stance phase data from all participants. NCC, NML, FPitch and the GRF (Rx, Ry, Rz) are provided in .mat files (Matlab). NCC and NML are denoted as NEle and NDev, respectively, in the data files. FPitch denotes the global plantar-/dorsiflexion angle (see also S1 Fig in the supporting information). One file with all steps under consideration is provided for each participant.
(ZIP)

S2 Data Oxford Foot Model stance phase data from all participants. Oxford Foot Model outputs are provided in .mat files (Matlab). One file with all steps under consideration is provided for each participant.
(ZIP)
