# Supplementary figures and images for: Cranio-caudal and medio-lateral navicular translation are representative surrogate measures of foot function in asymptomatic adults during walking
Source: PLoS One. 2018 Dec 5;13(12):e0208175. doi: 10.1371/journal.pone.0208175 (PMC6281217; doi:10.1371/journal.pone.0208175)

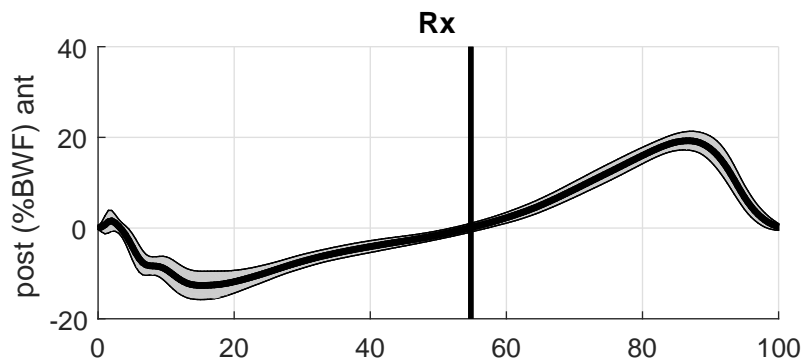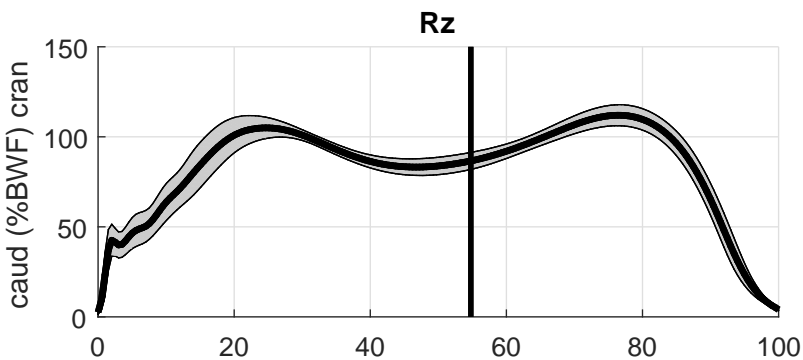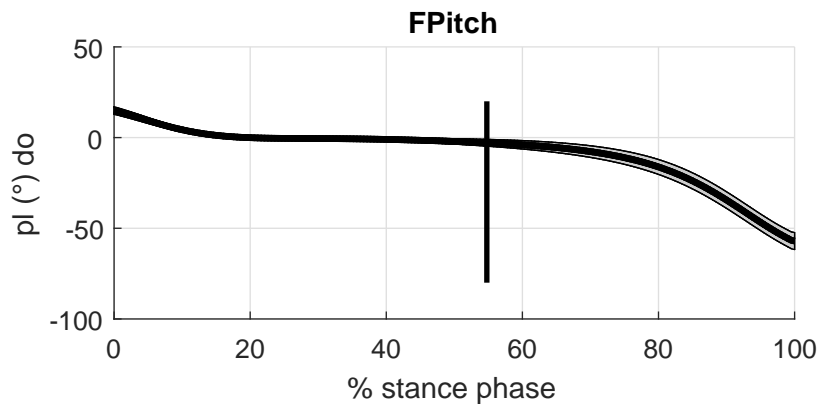

Supplement: S1 Fig — Means and standard deviations among participants (N = 19) of anterior-posterior (AP) and vertical ground reaction forces (Rx and Rz, respectively) normalized to the body weight force (BWF) and global plantar-/dorsiflexion angle of the foot (FPitch). The vertical lines indicate the transition of the AP ground reaction force from a posterior to an anterior direction (55 ± 2.7% stance phase) which was used to define the power absorption and power generation phases. This transition corresponds to the instance in time where lifting the heel from the ground was initiated. (PDF) [file pone.0208175.s001.pdf]
